# Supplementary material for: Distinct spin and orbital dynamics in Sr2RuO4
Source: Nat Commun. 2023 Nov 3;14:7042. doi: 10.1038/s41467-023-42804-3 (PMC10624926; doi:10.1038/s41467-023-42804-3)
Supplement: Supplementary file 1 — Supplementary Information [file 41467_2023_42804_MOESM1_ESM.pdf]

Supplementary Information for
Distinct spin and orbital dynamics in Sr_2RuO_4

H. Suzuki,[1,2,3] L. Wang,[1] J. Bertinshaw,[1] H. U. R. Strand,[4,5] S. Käser,[1,6] M. Krautloher,[1] Z. Yang,[1] N. Wentzell,[7] O. Parcollet,[7,8] F. Jerzembeck,[9] N. Kikugawa,[10] A. P. Mackenzie,[9] A. Georges,[7,11,12,13] P. Hansmann,[1,6,9] H. Gretarsson,[1,14] and B. Keimer[1]

[1]*Max-Planck-Institut für Festkörperforschung, Heisenbergstraße 1, D-70569 Stuttgart, Germany*
[2]*Frontier Research Institute for Interdisciplinary Sciences, Tohoku University, Sendai, 980-8578, Japan*
[3]*Institute of Multidisciplinary Research for Advanced Materials (IMRAM), Tohoku University, Sendai, 980-8578, Japan*
[4]*School of Science and Technology, Örebro University, Fakultetsgatan 1, SE-701 82, Örebro, Sweden*
[5]*Institute for Molecules and Materials, Radboud University, 6525 AJ Nijmegen, the Netherlands*
[6]*Department of Physics, Friedrich-Alexander-University (FAU) of Erlangen-Nürnberg, 91058 Erlangen, Germany*
[7]*Center for Computational Quantum Physics, Flatiron Institute, Simons Foundation, 162 5th Avenue, New York 10010, USA*
[8]*Université Paris-Saclay, CNRS, CEA, Institut de physique théorique, 91191, Gif-sur-Yvette, France*
[9]*Max Planck Institute for Chemical Physics of Solids, Nöthnitzer Straße 40, 01187 Dresden, Germany*
[10]*National Institute for Materials Science, Tsukuba, Ibaraki 305-0003, Japan*
[11]*Collége de France, 11 place Marcelin Berthelot, 75005 Paris, France*
[12]*Centre de Physique Théorique (CPHT), CNRS, Ecole Polytechnique, IP Paris, 91128 Palaiseau, France*
[13]*Department of Quantum Matter Physics, University of Geneva, 24 Quai Ernest-Ansermet, 1211 Geneva 4, Switzerland*
[14]*Deutsches Elektronen-Synchrotron DESY, Notkestraße 85, D-22607 Hamburg, Germany*

Supplementary Note 1: Incident energy dependence of RIXS spectra

Supplementary Fig. 1a shows the Ru L_3-edge x-ray absorption spectrum of Sr_2RuO_4 collected in the total fluorescence yield mode. The data were taken at the sample angle $\theta = 30^\circ$ at $T = 25$ K, with the π incident photon polarization. Two features observed at ~ 2838.9 and 2841.3 eV (blue triangles) correspond to the main transitions to the unoccupied $4d$ t_{2g} and e_g orbitals, respectively. We used 2838 eV (arrow), which is close to the Ru L_3-edge absorption threshold, for the RIXS measurements in the main text. Supplementary Fig. 1b shows a colormap of incident-energy dependence of RIXS intensity taken across the Ru L_3 edge. The data were taken with a low energy resolution setup ($\Delta E \sim 600$ meV). Close to the t_{2g} resonance (2838.9 eV), the feature below 1 eV is enhanced, whereas close to the e_g resonance (2841.3 eV) the feature around 3 eV is enhanced. This observation supports the assignment of the orbital characters of the RIXS features discussed in the main text. Above the e_g resonance, fluorescent-like signals (dashed lines) show up, whose energy loss evolves linearly with the incident energy. These fluorescent-like features originate from nonlocal excitations and bear resemblance to those observed in other perovskite ruthenates Ca_2RuO_4 [1] and $Ca_3Ru_2O_7$ [2].

Supplementary Note 2: Second derivative plot of RIXS intensity

The spin fluctuations along the $\mathbf{q} = (H,H)$ shown in Fig. 2a overlap with the low-energy tail of the orbital fluctuations with dominant spectral weight. Furthermore, the spectral weight of the spin fluctuations is supposed to be concentrated in the immediate vicinity of $\mathbf{q}_{\mathrm{ISF}}$ (see Supplementary Fig. 3). As a result, the spin fluctuations can be identified only as weak shoulder structures except near $\mathbf{q}_{\mathrm{ISF}}$. In addition, the theoretical DMFT+SOC susceptibilities (Supplementary Fig. 3) show that the RIXS spectral lineshape is neither represented by the Voigt functions nor by the damped harmonic oscillator functions, which are often used to fit the RIXS lineshapes from metallic systems. These situations make spectral fitting into multiple peaks numerically unstable and unjustified. To identify the location of peaks and shoulder structures of the spin fluctuations in an unbiased way, we plot the second derivative of the RIXS intensity with respect to energy in Supplementary Fig. 2. Along the (H,H) direction, the low-energy local maxima of the second derivative track the dispersion relation of the spin fluctuations (see also black circles in Fig. 2a), including the local energy minimum at $\mathbf{q}_{\mathrm{ISF}}$. The colormap also visualizes the presence of the weak spin fluctuations along the $(H,0)$ direction, with $\mathbf{q} = (-0.3,0)$ and $(-0.7,0)$. A weak orbital fluctuation branch which disperses from ~ 0.2 eV at $(0,0)$ to ~ 0.3 eV at $(-0.5,0)$ is also identified, which corresponds to the $\langle L_z L_z \rangle$ component of the orbital dynamical response function (see Supplementary Fig. 3).

Supplementary Note 3: Dynamical response functions

The theoretical calculation of the spin and orbital angular momentum susceptibilities $\chi_{S_\mu S_\mu}$ and $\chi_{L_\mu L_\mu}$ with Ru $4d-t_{2g}$ symmetry was performed by solving the Bethe-Salpeter equation with the dynamical mean field theory (DMFT) [3] approximation for the particle hole irreducible vertex [4–7] and a bare generalized susceptibility χ_0 containing both DMFT self-energy and correlation enhanced spin-orbit coupling (SOC) corrections [8, 9]. The calculations were performed using the two-particle response function toolbox (TPRF) [10] in the imaginary time formalism and analytically continued to real frequency with the maximum entropy algorithm [11] using the `ana_cont` package [12] and 12 sampled bosonic Matsubara frequencies. The vertex and self-energy was computed within DMFT without SOC at the temperature 386 K, due to technical limitations in the hybridization expansion [13–16] continuous time quantum Monte Carlo impurity solver [17]. The effective low energy model was constructed by combining, i) maximally localized Wannier functions using Wannier90 [18–20] and Wien2Wannier [21] for the three bands crossing the Fermi level with Ru t_{2g} symmetry, and ii) a local Kanamori interaction [22] with a Hubbard $U = 2.3\,\text{eV}$ and a Hund's coupling $J = 0.4\,\text{eV}$ [23]. The Wannier construction was performed with an energy window of $[-2.85, 0.75]\,\text{eV}$ on the band structure from a density functional theory calculation of Sr_2RuO_4 using the PBE density functional [24] and Wien2k [25] with a 20^3 k-point grid and the experimental crystal structure (at 100 K) [26]. The effective model is identical to the one used in [8, 9]. All calculations were built using the toolbox for interacting quantum systems (TRIQS) [27]. The resulting DMFT+SOC susceptibility components $\chi_{S_\mu S_\mu}$ and $\chi_{L_\mu L_\mu}$ (Supplementary Fig. 3) display the energy scale separation between the spin fluctuations at energies $\sim 0.1\,\text{eV}$ and the orbital fluctuations at energies $\gtrsim 0.2\,\text{eV}$ with the in-plane response (xx and yy) peaking at $\sim 0.75\,\text{eV}$ while the out-of-plane response peaking at $\sim 0.4\,\text{eV}$. Supplementary Fig. 4 shows the effect of neglecting SOC in the bare susceptibility. The magnitude of all components is increased, in particular the incommensurate spin peak at $(H,H) \sim (0.3, 0.3)$, and the spin susceptibility disperses more strongly down in energy at $(0,0)$. Neglecting the dynamical vertex corrections in DMFT results in the random phase approximation (RPA) with only static interactions and the resulting susceptibilities do not display the spin and orbital angular-momentum energy scale separation, see Supplementary Fig. 5. Static screening was accounted for in the RPA calculation by reducing the local interaction to $U = 0.575\,\text{eV}$ and $J = 0.1\,\text{eV}$ keeping the J/U ratio fixed [9]. Neglecting interactions all together gives the bare susceptibility, which even lacks the low energy incommensurate spin fluctuations, see Supplementary Fig. 6.

Supplementary Note 4: Fitting of RIXS intensity based on theoretical susceptibilities

With the theoretical spin and orbital susceptibilities at hand, we have constructed theoretical RIXS intensity in the following way. In general, the RIXS cross section is given by the Kramers-Heisenberg formula [28]:

$$\frac{d^2\sigma}{d\Omega d\omega} \propto \sum_f \left| \langle f | T^\dagger_{\varepsilon_o} \frac{1}{\omega_i + E_i + i\Gamma - H} T_{\varepsilon_i} | i \rangle \right|^2 \delta(\omega_i - \omega_o + E_i - E_f), \tag{1}$$

where H is the Hamiltonian and E_i (E_f) is the energy of the initial state $|i\rangle$ (final state $|f\rangle$). ω_i and ε_i (ω_o and ε_o) are the energy and polarization of the incoming (outgoing) photons. $T_\varepsilon = \mathbf{p}\cdot\mathbf{A}$ is the optical transition operator, which is expressed as a summation of local transition operators at site j: $T_\varepsilon = \sum_j e^{i\mathbf{k}\cdot\mathbf{r}_j} T_{j,\varepsilon}$. As the core hole is created and annihilated at the same site, the total RIXS transition operator $R^{\varepsilon_i\varepsilon_o} = T^\dagger_{\varepsilon_o} \frac{1}{\omega_i+E_i+i\Gamma-H} T_{\varepsilon_i}$ is expressed as a Fourier transform of local RIXS operators:

$$\begin{aligned} R^{\varepsilon_i\varepsilon_o} &= \sum_{j',j} e^{i(\mathbf{k}_i\cdot\mathbf{r}_j - \mathbf{k}_o\cdot\mathbf{r}_{j'})} T^\dagger_{j',\varepsilon_o} \frac{1}{\omega_i + E_i + i\Gamma - H} T_{j,\varepsilon_i} \\ &= \sum_j e^{i\mathbf{Q}\cdot\mathbf{r}_j} T^\dagger_{j,\varepsilon_o} \frac{1}{\omega_i + E_i + i\Gamma - H} T_{j,\varepsilon_i} \\ &= \sum_j e^{i\mathbf{Q}\cdot\mathbf{r}_j} R_j^{\varepsilon_i\varepsilon_o} && (2) \\ &= R_{\mathbf{Q}}^{\varepsilon_i\varepsilon_o}, && (3) \end{aligned}$$

where $\mathbf{Q} = \mathbf{k}_i - \mathbf{k}_o$ is the momentum transfer to the sample. The momentum transfer in the main text is expressed with its in-plane component $\mathbf{q}$.

As the terms included in $R_j^{\varepsilon_i\varepsilon_o}$, we consider only the on-site operators at site j and neglect operators involving the neighboring sites. Furthermore, we employ cubic crystal field symmetry (O_h) around the Ru ion at site j. Then $R_j^{\varepsilon_i\varepsilon_o}$, a bilinear of the components of the polarization vectors ε_o^* and ε_i, can be decomposed into basis operators of different irreducible representations of the O_h point group:

$$R_j^{\varepsilon_i\varepsilon_o} = (\varepsilon_o^* \cdot \varepsilon_i) O_j^{A_{1g}} + (\varepsilon_o^* \times \varepsilon_i) \cdot \mathbf{N}_j^{T_{1g}} + \sum_\Gamma [\varepsilon_o^*, \varepsilon_i]^\Gamma \cdot \Xi_j^\Gamma, \tag{4}$$

where Γ represents T_{2g} or E_g. $[\varepsilon_o^*, \varepsilon_i]^\Gamma$ is the basis of the irreducible representation Γ composed of the polarization vectors ε_o^* and ε_i. $O_j^{A_{1g}}, \mathbf{N}_j^{T_{1g}}, \Xi_j^\Gamma$ represent the A_{1g} (scalar), T_{1g} (pseudovector), and Γ (quadrupolar) operators, respectively. The dot product in the last term represents the symmetric contraction of indices. In the present 90° scattering geometry (see Fig. 1a), $\varepsilon_o^* \perp \varepsilon_i$ holds regardless of the measured $\mathbf{Q}$. This condition suppresses the scalar (charge) transitions and enhances the pseudovector (magnetic) transitions. We therefore consider only the magnetic channel in the theoretical treatment below. Note, however, that the quadrupolar transitions remain finite.

The orbital degrees freedom in the t_{2g}^4 electron configurations of Sr_2RuO_4, combined with the spin degrees of freedom ($L = 1, S = 1$), allow a variety of terms in the magnetic channel from a symmetry point of view. This is readily observed in the analytical form of $\mathbf{N}_j^{T_{1g}}$ under the fast-collision approximation [29], which includes several combinations of the spin ($\mathbf{S}_j$) and orbital angular momentum ($\mathbf{L}_j$) operators. Here, for simplicity, we approximate $\mathbf{N}_j^{T_{1g}}$ as a linear combination of spin and orbital angular momentum operators:

$$\mathbf{N}_j^{T_{1g}} = \mathbf{S}_j + \alpha \mathbf{L}_j, \tag{5}$$

where α is a fitting parameter accounting for the relative ratio, which varies as a function of the incident x-ray energy (see Supplementary Fig. 1b).

Under this approximation, the theoretical RIXS intensity is expressed as

$$\begin{aligned} I(\mathbf{Q}, \omega) &= -\frac{1}{\pi} \mathrm{Im} \left\langle R_{\mathbf{Q}}^{\varepsilon_i\varepsilon_o \dagger} R_{\mathbf{Q}}^{\varepsilon_i\varepsilon_o} \right\rangle \\ &= -\frac{1}{\pi} \mathrm{Im} \left\langle R_{-\mathbf{Q}}^{\varepsilon_i\varepsilon_o} R_{\mathbf{Q}}^{\varepsilon_i\varepsilon_o} \right\rangle, \end{aligned} \tag{6}$$

with

$$R_{\mathbf{Q}}^{\varepsilon_i\varepsilon_o} = \varepsilon_o^* \times \varepsilon_i \cdot (\mathbf{S}_{\mathbf{Q}} + \alpha \mathbf{L}_{\mathbf{Q}}). \tag{7}$$

The polarization vectors ε_o^* and ε_i are real-valued in the present case (linear polarizations) and vary with the measured $\mathbf{Q}$. The RIXS intensity is thus given by a linear combination of the correlation functions of the $S_{\mathbf{Q}}^\mu$ and $L_{\mathbf{Q}}^\mu$ ($\mu = x, y, z$) operators. It is found that the cross terms $\left\langle S_{-\mathbf{Q}}^\mu L_{\mathbf{Q}}^\nu \right\rangle$, and the correlators with different indices $\left\langle S_{-\mathbf{Q}}^\mu S_{\mathbf{Q}}^\nu \right\rangle$ and $\left\langle L_{-\mathbf{Q}}^\mu L_{\mathbf{Q}}^\nu \right\rangle$ ($\mu \neq \nu$), either vanish by symmetry or are negligibly small. We therefore consider contributions from $\left\langle S_{-\mathbf{Q}}^\mu S_{\mathbf{Q}}^\mu \right\rangle$ and $\left\langle L_{-\mathbf{Q}}^\mu L_{\mathbf{Q}}^\mu \right\rangle$ to the RIXS intensity [Eq. (6)]. To best reproduce the experimental RIXS intensity for each theoretical approximation, the fitting parameter α is set to 3.6 for DMFT+SOC and DMFT susceptibilities, and to 1.3 for RPA and bare susceptibilities. The theoretical RIXS intensities for all these approximations are summarized in Supplementary Fig. 7. Note that $\alpha = 3.6$ for DMFT+SOC and DMFT enhances the contributions from the orbital susceptibilities by a factor of ~ 13. This is due to the reduced intensity scale of the orbital susceptibilities compared to the spin susceptibilities (see the color bars in Supplementary Fig. 3).

Here we provide qualitative reasoning behind the success of this simple approach. Our approximation Eq. (5) is the simplest linear combination of $\mathbf{S}$ and $\mathbf{L}$ that transforms as a pseudovector. On the other hand, the analytic expression for the L_3-edge magnetic transition operator $\mathbf{N}$ for a t_{2g}^4 ion within the fast collision approximation, apart from a constant prefactor, is [29]

$$N_z = 2L_z - 4L_z^2 S_z + L_z(\mathbf{L} \cdot \mathbf{S}) + (\mathbf{L} \cdot \mathbf{S}) L_z. \tag{8}$$

N_x and N_y follow from symmetry. While the $\mathbf{L}$ operator appears as it is (first term), the $\mathbf{S}$ operator is coupled with quadratic terms of $\mathbf{L}$ operators (last three terms). In Sr_2RuO_4, the spin and orbital fluctuations are energetically separated (Supplementary Fig. 3). Therefore, spin excitation intensity is suppressed in the RIXS cross section. Correspondingly, we need to enhance the $\mathbf{L}$ term in the fit ($\alpha = 3.6$).

Supplementary Note 5: Origin of 0.5 eV peak around zone center

Here we discuss possible origins of the broad 0.5 eV peak observed around $\mathbf{q} = (0, 0)$, which does not have a corresponding branch in the theoretical RIXS intensity (Fig. 3b). We note that the present theoretical model focuses on the electronic degrees of

freedom within the t_{2g} orbitals. It is therefore possible that the inclusion of the e_g orbitals to the Hamiltonian and the hybridization between the t_{2g} and e_g orbitals could generate this feature, in addition to the dispersive feature C (Fig. 1c). Moreover, a coupling to lattice degrees of freedom could yield additional structures. Finally, we note that the calculation was done at $T \approx 386$ K well above the temperature of the experiment at 25 K (i.e. in the Fermi liquid regime). While such a high calculation temperature would prohibit comparison e.g. to low temperature transport, we do not expect significant temperature effects in the RIXS spectra at finite frequencies.

Furthermore, the RIXS cross section in the present geometry includes not only the magnetic responses but also the quadrupolar responses, which originate from the last term in eq. (4) and are neglected in the treatment above. The quadrupolar transitions have indeed been observed in a d^4 cubic ruthenium Mott insulator K_2RuCl_6 [30], where spin-orbit transitions from the $J = 0$ nonmagnetic ground state to the $J = 2$ quadrupolar states are located above the main transitions to the $J = 1$ magnetic states. The intensity of the $J = 2$ transitions is about half of that of the $J = 1$ transitions. This suggests that the quadrupolar operators generally remain active in the Ru L_3 RIXS process in the d^4 ruthenium compounds with octahedral crystal field environment. Thus, the quadrupolar transitions could contribute to the broad continuum around $\mathbf{q} = (0,0)$, which is less pronounced than the main orbital response at 0.2 eV.

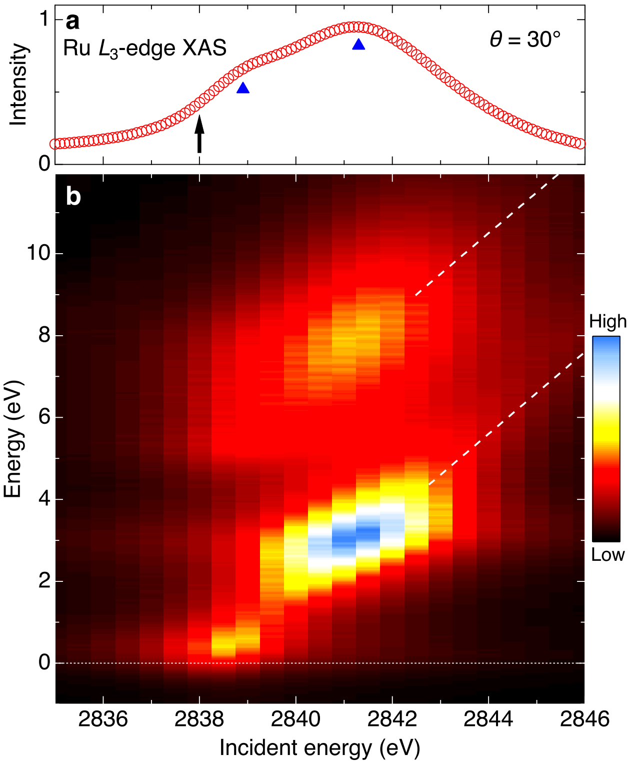

Supplementary Fig. 1. Incident energy dependence of RIXS spectra. a, X-ray absorption spectrum of Sr_2RuO_4 around the Ru L_3 edge. The blue triangles indicate the main transitions to the unoccupied Ru 4d t_{2g} and e_g orbitals, respectively. The arrow indicates the incident energy (2838 eV) used for the RIXS measurements in the main text. **b**, Colormap of the incident-energy dependence of the RIXS spectra across the Ru L_3 edge, taken with a low-resolution setup ($\Delta E \sim 600$ meV). The diagonal dotted lines are guides to the eye representing the fluorescent emission. All the data are taken with the incident angle of $\theta = 30^\circ$ at $T = 25$ K.

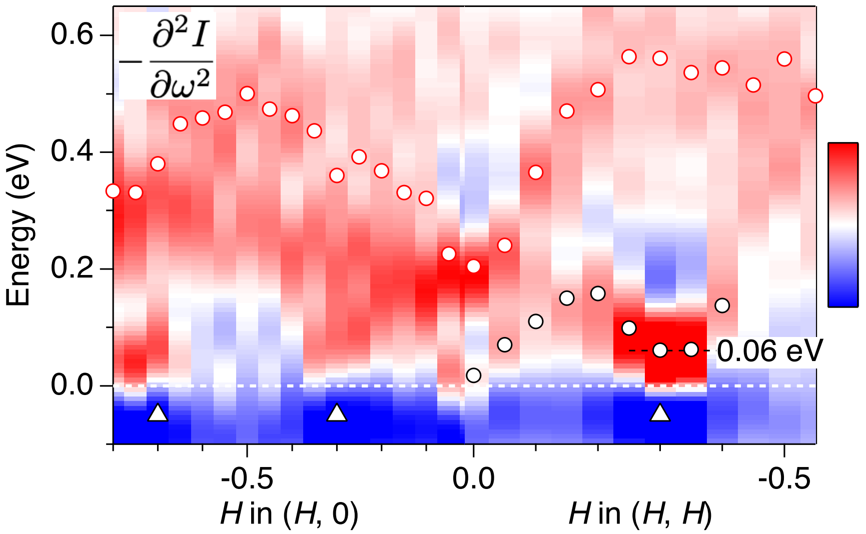

Supplementary Fig. 2. Second derivative plot of the RIXS intensity. Second derivative of RIXS intensity with respect to the energy loss. The **q** positions of low-energy spin fluctuations are indicated with triangles. Black circles along the $\mathbf{q} = (H, H)$ direction indicate the local maxima of the second derivative, which are associated with the dispersion of the spin fluctuations. The global peak maxima of the original RIXS intensity from orbital fluctuations are also plotted with red circles.

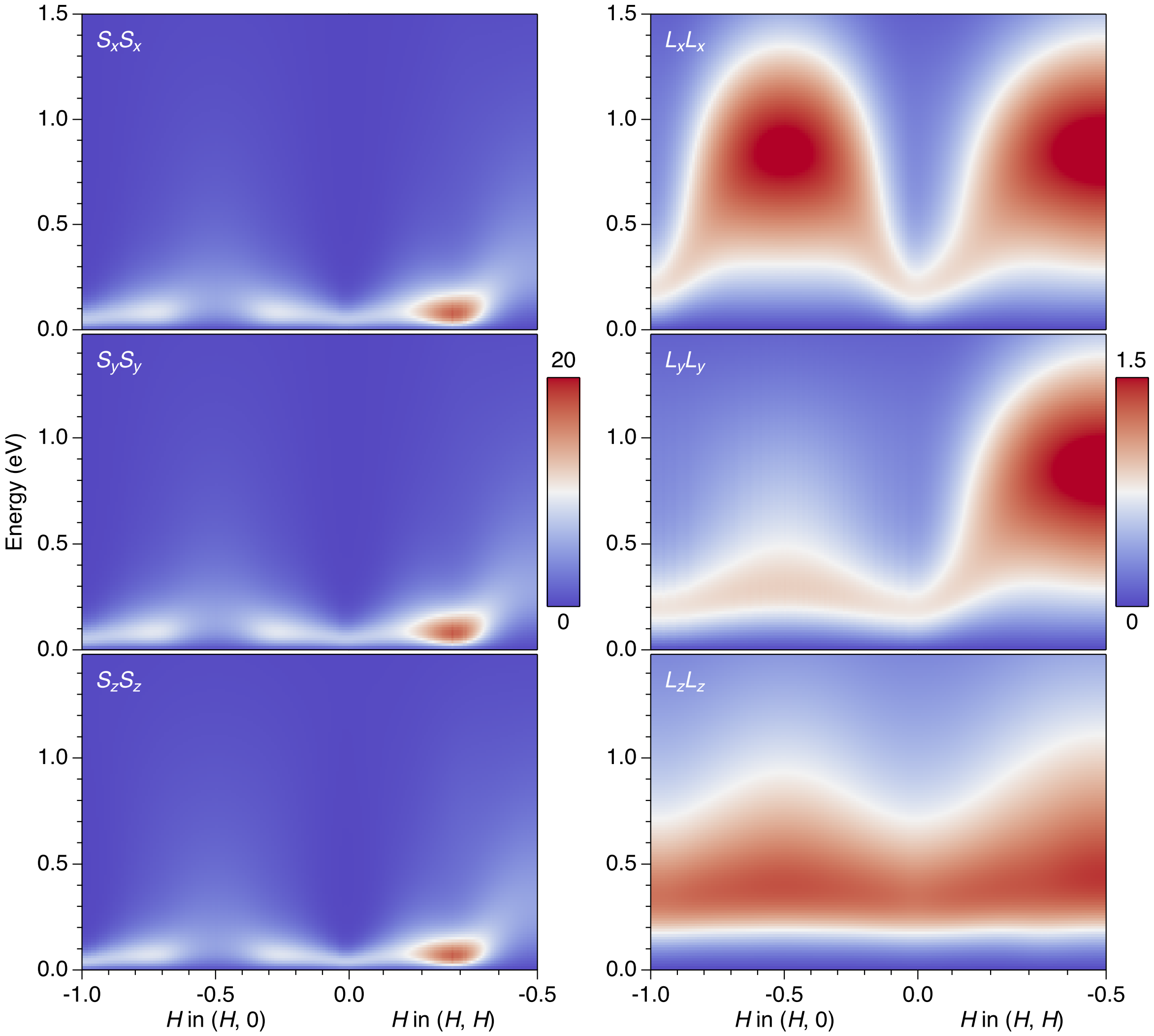

Supplementary Fig. 3. DMFT+SOC susceptibilities. Theoretical dynamical mean field theory and spin-orbit coupling (DMFT+SOC) spin and orbital angular-momentum susceptibilities $\chi_{S_\mu S_\mu}$ and $\chi_{L_\mu L_\mu}$ in the plane of the momentum paths $(H,0)$ and (H,H) and energy.

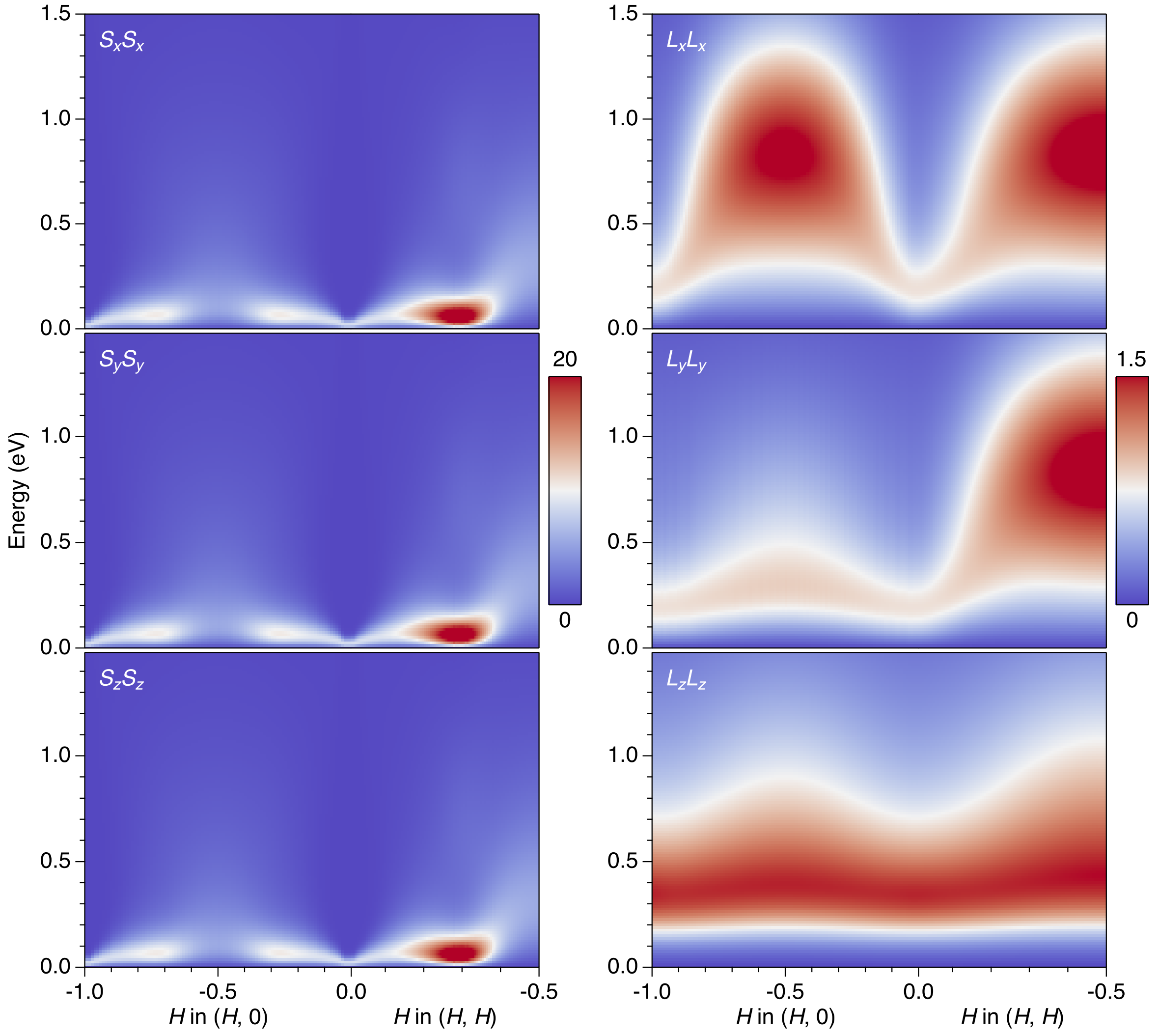

Supplementary Fig. 4. DMFT susceptibilities. Theoretical dynamical mean field theory (DMFT) spin and orbital angular-momentum susceptibilities $\chi_{S_\mu S_\mu}$ and $\chi_{L_\mu L_\mu}$ in the plane of the momentum paths $(H,0)$ and (H,H) and energy, showing the result of neglecting spin-orbit coupling (SOC), c.f. Supplementary Fig. 3.

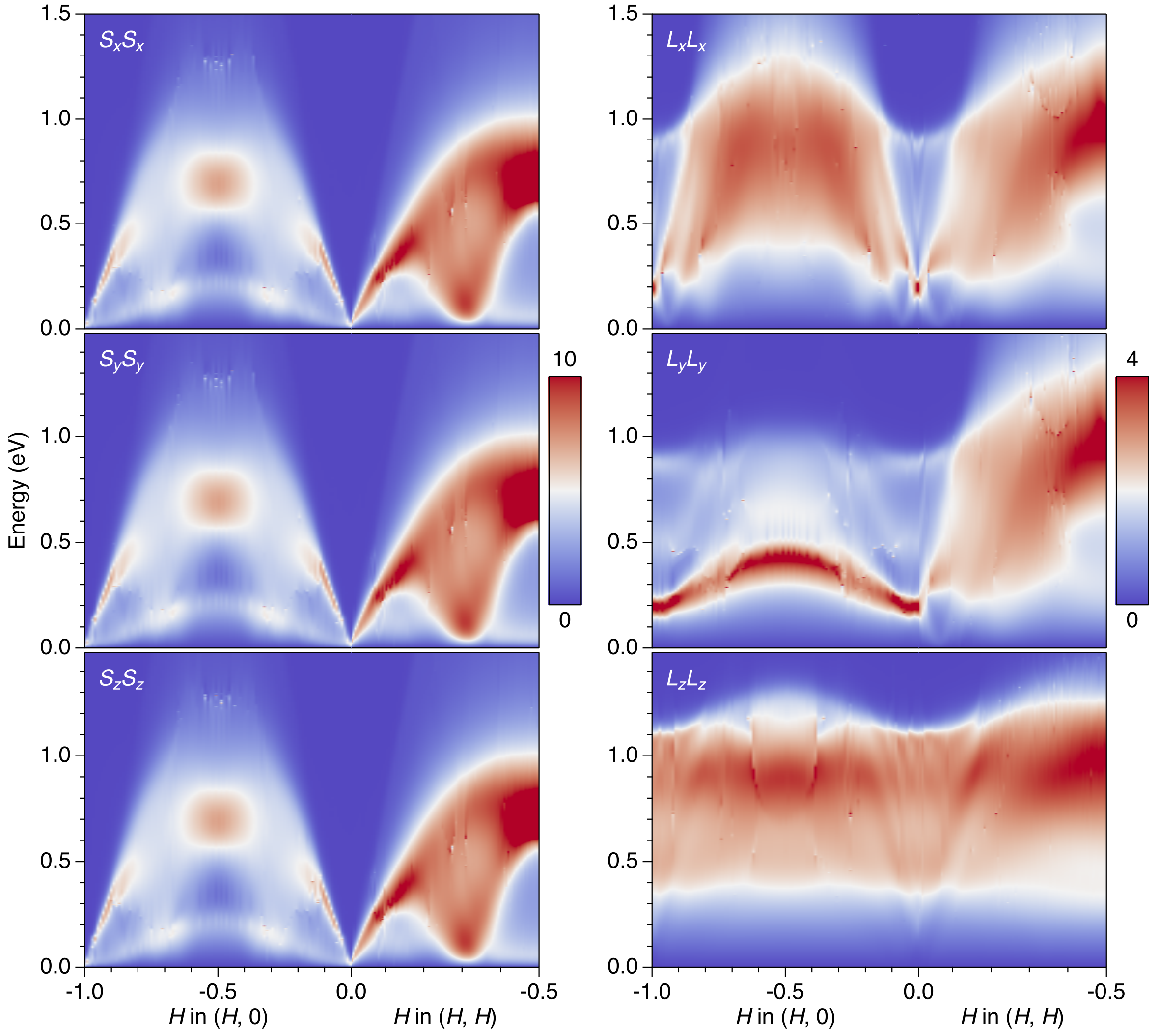

Supplementary Fig. 5. RPA susceptibilities. Theoretical random phase approximation (RPA) spin and orbital angular-momentum susceptibilities $\chi_{S_\mu S_\mu}$ and $\chi_{L_\mu L_\mu}$ in the plane of the momentum paths $(H,0)$ and (H,H) and energy, showing the result of neglecting dynamical vertex corrections, c.f. Supplementary Fig. 4.

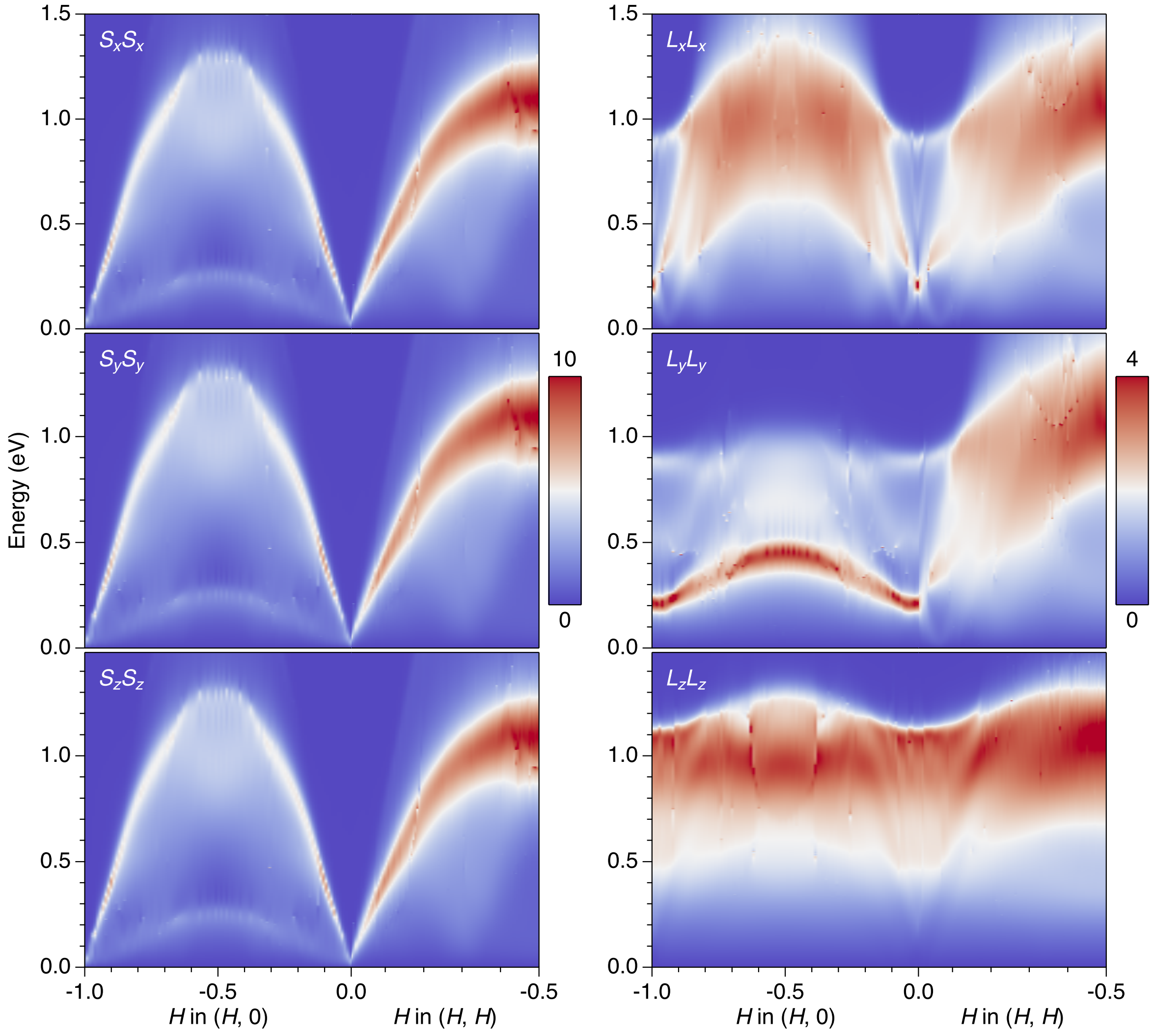

Supplementary Fig. 6. Bare susceptibilities. Theoretical *bare* spin and orbital angular-momentum susceptibilities $\chi_{S_\mu S_\mu}$ and $\chi_{L_\mu L_\mu}$ in the plane of the momentum paths $(H,0)$ and (H,H) and energy, showing the result of entirely neglecting interactions on the two-particle level, c.f. RPA in Supplementary Fig. 5 and DMFT in Supplementary Fig. 4.

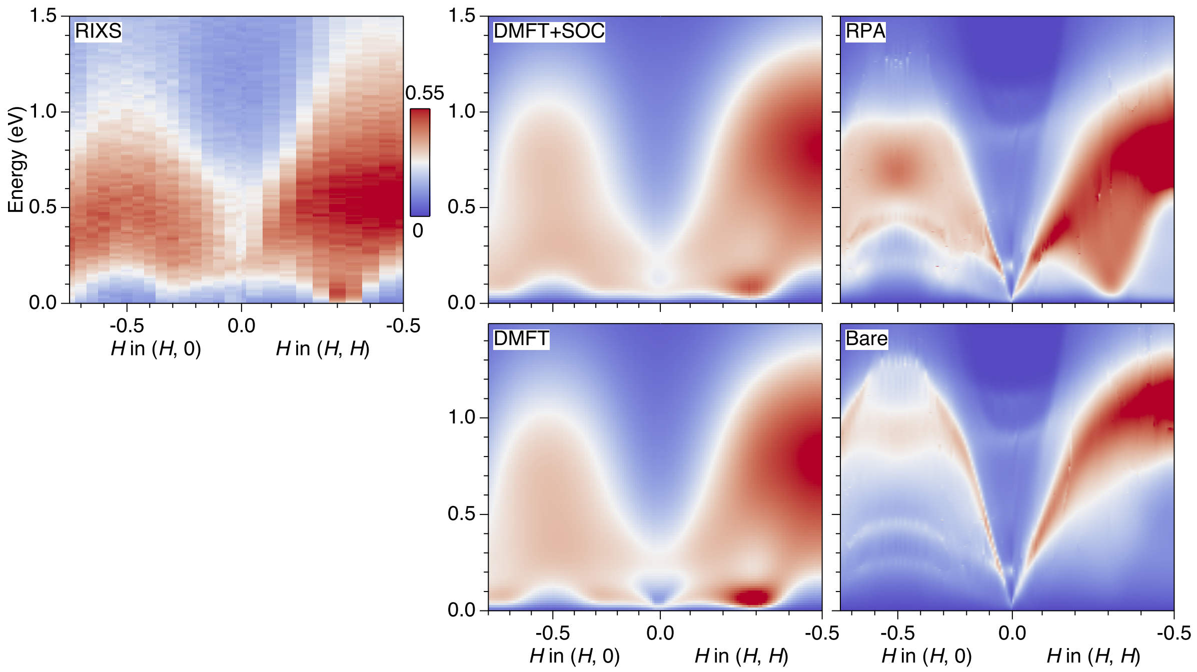

Supplementary Fig. 7. RIXS intensity simulations based on different theoretical approximations. Comparison between the experimental RIXS spectra **(RIXS)** and the theoretically computed RIXS spectra from DMFT including SOC **(DMFT+SOC)**. Lower levels of theory, such as DMFT without SOC **(DMFT)**, RPA without vertex corrections **(RPA)**, and the bare susceptibility without interactions on the two-particle level **(Bare)**, differ qualitatively from the experimental result.

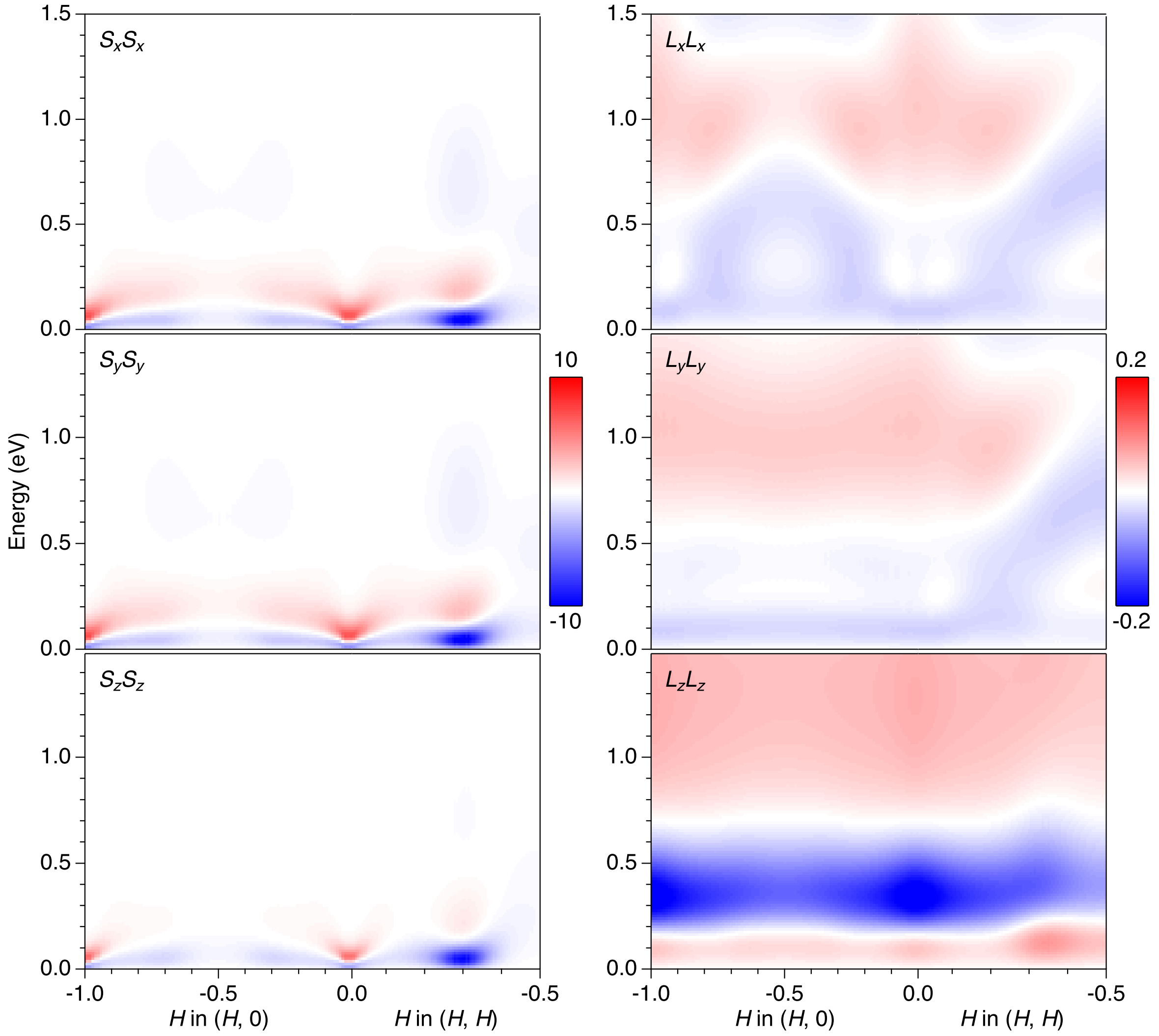

Supplementary Fig. 8. Effect of SOC on the DMFT susceptibilities. Difference plot between the DMFT+SOC and DMFT susceptibilities.

[1] Gretarsson, H. et al. Observation of spin-orbit excitations and Hund's multiplets in Ca_2RuO_4. *Phys. Rev. B* **100**, 045123 (2019).
[2] Bertinshaw, J. et al. Spin and charge excitations in the correlated multiband metal $Ca_3Ru_2O_7$. *Phys. Rev. B* **103**, 085108 (2021).
[3] Georges, A., Kotliar, G., Krauth, W. & Rozenberg, M. J. Dynamical mean-field theory of strongly correlated fermion systems and the limit of infinite dimensions. *Rev. Mod. Phys.* **68**, 13–125 (1996).
[4] Jarrell, M. Hubbard model in infinite dimensions: A quantum Monte Carlo study. *Phys. Rev. Lett.* **69**, 168–171 (1992).
[5] Kuneš, J. Efficient treatment of two-particle vertices in dynamical mean-field theory. *Phys. Rev. B* **83**, 085102 (2011).
[6] Boehnke, L., Hafermann, H., Ferrero, M., Lechermann, F. & Parcollet, O. Orthogonal polynomial representation of imaginary-time Green's functions. *Phys. Rev. B* **84**, 075145 (2011).
[7] Lin, N., Gull, E. & Millis, A. J. Two-particle response in cluster dynamical mean-field theory: Formalism and application to the Raman response of high-temperature superconductors. *Phys. Rev. Lett.* **109**, 106401 (2012).
[8] Tamai, A. et al. High-resolution photoemission on Sr_2RuO_4 reveals correlation-enhanced effective spin-orbit coupling and dominantly local self-energies. *Phys. Rev. X* **9**, 021048 (2019).
[9] Strand, H. U. R., Zingl, M., Wentzell, N., Parcollet, O. & Georges, A. Magnetic response of Sr_2RuO_4: Quasi-local spin fluctuations due to Hund's coupling. *Phys. Rev. B* **100**, 125120 (2019).
[10] Strand, H. U. R. Two-particle response function tool-box (tprf) for TRIQS. *github.com/TRIQS/tprf* (2019).
[11] Jarrell, M. & Gubernatis, J. Bayesian inference and the analytic continuation of imaginary-time quantum Monte Carlo data. *Phys. Rep.*

269, 133–195 (1996).
[12] Kaufmann, J. & Held, K. https://arxiv.org/abs/2105.11211, (2021).
[13] Werner, P., Comanac, A., de' Medici, L., Troyer, M. & Millis, A. J. Continuous-time solver for quantum impurity models. *Phys. Rev. Lett.* **97**, 076405 (2006).
[14] Werner, P. & Millis, A. J. Hybridization expansion impurity solver: General formulation and application to Kondo lattice and two-orbital models. *Phys. Rev. B* **74**, 155107 (2006).
[15] Haule, K. Quantum Monte Carlo impurity solver for cluster dynamical mean-field theory and electronic structure calculations with adjustable cluster base. *Phys. Rev. B* **75**, 155113 (2007).
[16] Gull, E. et al. Continuous-time Monte Carlo methods for quantum impurity models. *Rev. Mod. Phys.* **83**, 349–404 (2011).
[17] Seth, P., Krivenko, I., Ferrero, M. & Parcollet, O. TRIQS/cthyb: A continuous-time quantum Monte Carlo hybridisation expansion solver for quantum impurity problems. *Comput. Phys. Commun.* **200**, 274–284 (2016).
[18] Marzari, N. & Vanderbilt, D. Maximally localized generalized Wannier functions for composite energy bands. *Phys. Rev. B* **56**, 12847–12865 (1997).
[19] Mostofi, A. A. et al. wannier90: A tool for obtaining maximally-localised wannier functions. *Comput. Phys. Commun.* **178**, 685 – 699 (2008).
[20] Marzari, N., Mostofi, A. A., Yates, J. R., Souza, I. & Vanderbilt, D. Maximally localized Wannier functions: Theory and applications. *Rev. Mod. Phys.* **84**, 1419–1475 (2012).
[21] Kuneš, J. et al. Wien2wannier: From linearized augmented plane waves to maximally localized Wannier functions. *Comput. Phys. Commun.* **181**, 1888–1895 (2010).
[22] Kanamori, J. Electron correlation and ferromagnetism of transition metals. *Prog. Theor. Phys.* **30**, 275–289 (1963).
[23] Mravlje, J. et al. Coherence-incoherence crossover and the mass-renormalization puzzles in Sr_2RuO_4. *Phys. Rev. Lett.* **106**, 096401 (2011).
[24] Perdew, J. P., Burke, K. & Ernzerhof, M. Generalized gradient approximation made simple. *Phys. Rev. Lett.* **77**, 3865–3868 (1996).
[25] Blaha, P. et al. *WIEN2k, An Augmented Plane Wave + Local Orbitals Program for Calculating Crystal Properties*. Karlheinz Schwarz, Techn. Universität Wien, Austria, (2018).
[26] Vogt, T. & Buttrey, D. J. Low-temperature structural behavior of Sr_2RuO_4. *Phys. Rev. B* **52**, R9843–R9846 (1995).
[27] Parcollet, O. et al. TRIQS: A toolbox for research on interacting quantum systems. *Comput. Phys. Commun.* **196**, 398–415 (2015).
[28] Haverkort, M. W. Theory of resonant inelastic x-ray scattering by collective magnetic excitations. *Phys. Rev. Lett.* **105**, 167404 (2010).
[29] Kim, B. J. & Khaliullin, G. Resonant inelastic x-ray scattering operators for t_{2g} orbital systems. *Phys. Rev. B* **96**, 085108 (2017).
[30] Takahashi, H. et al. Nonmagnetic $J = 0$ state and spin-orbit excitations in K_2RuCl_6. *Phys. Rev. Lett.* **127**, 227201 (2021).
